# Supplementary material for: Enhanced formation of methane hydrate from active ice with high gas uptake
Source: Nat Commun. 2023 Dec 6;14:8068. doi: 10.1038/s41467-023-43487-6 (PMC10700304; doi:10.1038/s41467-023-43487-6)
Supplement: Supplementary file 1 — Supplementary information [file 41467_2023_43487_MOESM1_ESM.pdf]

Supplementary Information for

# Enhanced formation of methane hydrate from active ice with high gas uptake

Peng Xiao[1], Juan-Juan Li[1], Wan Chen[1], Wei-Xin Pang[2], Xiao-Wan Peng[1], Yan Xie[1], Xiao-Hui Wang[1], Chun Deng[1], Chang-Yu Sun[1], Bei Liu[1], Yu-Jie Zhu[1], Yun-Lei Peng[1], Praveen Linga[3], Guang-Jin Chen[1]

[1] State Key Laboratory of Heavy Oil Processing, China University of Petroleum, Beijing 102249, P. R. China.

[2] State Key Laboratory of Natural Gas Hydrate, CNOOC Research Institute Co., Ltd., Beijing 100027, China.

[3] Department of Chemical and Biomolecular Engineering, National University of Singapore; Singapore 117585, Singapore.

Correspondence to: cysun@cup.edu.cn (C. Y. Sun); liub@cup.edu.cn (B. Liu); chepl@nus.edu.sg (P. Linga), gjchen@cup.edu.cn (G. J. Chen)

**This Supplementary Information includes:**

**Supplementary methods**

**Ⅰ. Data Treatment**

**Ⅱ. Characterization**

**Supplementary Figures 1 to 16**

**Supplementary Tables 1 to 3**

# Supplementary Methods

## Ⅰ. *Data treatment*

In the gas storage tests, the number of moles of gas fixed in the hydrate was calculated based on the molar balance of gas in the gas and hydrate phase in the gas reservoir and the reactor. At time *t*, the balance can be expressed as

$$N_{\mathrm{in}} = N_{\mathrm{gas},t} + N_{\mathrm{hydrate},t} \tag{S1}$$

where $N_{\mathrm{hydrate,t}}$ is the number of moles of gas stored in gas hydrate at time *t*. $N_{\mathrm{in}}$ is the number of moles of gas injected into the reactor, which was calculated by

$$N_{\mathrm{in}} = \frac{P_{\mathrm{a},0}V_{\mathrm{a}}}{Z_{\mathrm{a},0}RT} - \frac{P_{\mathrm{a},t}V_{\mathrm{a}}}{Z_{\mathrm{a},t}RT} \tag{S2}$$

$N_{\mathrm{gas,t}}$ represents the number of moles of remained gas in the reactor at time *t*; it was calculated by

$$N_{\mathrm{gas},t} = \frac{P_{\mathrm{gas},t}V_{\mathrm{gas},t}}{Z_{\mathrm{gas},t}RT} \tag{S3}$$

In equations (S2) and (S3), the subscript a represents the gas reservoir; 0 refers to the stable state of the gas in the gas reservoir before injecting gas into the reactor; subscript gas refers to the gas phase in the reactor. *Z* is the compressibility factor, which was calculated by the BWRS equation of state. The number of moles of gas stored in gas hydrate was calculated by

$$N_{\mathrm{hydrate},t} = \frac{m_{\mathrm{w}}x_t}{18\times 6} \tag{S4}$$

where $m_{\mathrm{w}}$ is the initial mass of water (ice). $x_t$ is the ratio of ice converted to gas hydrate. 18 is the molar mass of water. The hydration number was set to 6.0 in this work. Since the temperature for hydrate formation was below the ice point, no obvious liquid water was left in the reactor; thus, the volume of the free gas in the reactor was calculated by

$$V_{\mathrm{gas},t} = V_{\mathrm{r}} - V_{\mathrm{stirrer}} - 1.25V_{\mathrm{w}}x_t - 1.087V_{\mathrm{w}}(1-x_t) \tag{S5}$$

where $V_{\mathrm{r}}$ represents the internal volume of the sapphire reactor, $\mathrm{m}^3$; $V_{\mathrm{stirrer}}$ is the volume of the stirrer; 1.25 and 1.087 are the expansion factors of hydrate and ice compared to water. $V_{\mathrm{w}}$ is the original volume of water, which was calculated by initial

mass of water $m_w$ and the density of $1\times10^6$ g m$^{-3}$. Combing equations (S1) ~ (S5), $x_t$ can be expressed as

$$x_t = \left[ N_{\mathrm{in},t} - \frac{P_{\mathrm{gas},t}(V_\mathrm{b} - V_\mathrm{stirrer} - 1.087V_\mathrm{w})}{Z_{\mathrm{gas},t}RT} \right] \Big/ \left(\frac{m_\mathrm{w}}{108} - \frac{0.163P_{\mathrm{gas},t}V_\mathrm{w}}{Z_{\mathrm{gas},t}RT}\right) \tag{S6}$$

Then, $N_{\mathrm{hydrate,t}}$ was calculated by equation (S4). The gas uptake (GU, $V_\mathrm{g}$ $V_\mathrm{w}^{-1}$) was calculated by

$$\mathrm{GU} = 0.0224N_{\mathrm{hydrate},t} / V_\mathrm{w} \tag{S7}$$

Methane generally forms structure Ⅰ hydrate, and for the ideal situation that each hydrate cage is occupied by one methane molecule, the ideal value of GU is 216. The storage density (SD, $V_\mathrm{g}$ $V_\mathrm{bed}^{-1}$) of gas in the hydrate was calculated by

$$\mathrm{SD} = 0.0224N_{\mathrm{hydrate},t} / V_\mathrm{bed} \tag{S8}$$

where $V_\mathrm{bed}$ represents the apparent volume of the hydrate bed formed in the reactor. It was calculated by

$$V_\mathrm{bed} = S_\mathrm{r} \times H_\mathrm{bed} \tag{S9}$$

where $S_\mathrm{r}$ is the inner cross section area of the reactor; $H_\mathrm{bed}$ is the height of the hydrate bed.

In the gas separation test, the separation factor of component 2 over component 1 was calculated by

$$\beta = \frac{x_2/y_2}{x_1/y_1} \tag{S10}$$

where $x_i$ and $y_i$ are the molar fractions of component $i$ in the hydrate and gas phase, respectively. $y_i$ was determined with gas chromatography. $x_i$ was calculated based on the following equations

$$N_\mathrm{in}z_1 = N_{\mathrm{gas},t}y_1 + N_{\mathrm{hydrate},t}x_1 \tag{S11}$$

$$N_\mathrm{in}z_2 = N_{\mathrm{gas},t}y_2 + N_{\mathrm{hydrate},t}x_2 \tag{S12}$$

where $z_1$ and $z_2$ are the molar fractions of component 1 and 2 in the feed gas, respectively. $z_1$, $z_2$ were determined with gas chromatography. In equations (S11) and S12, $N_\mathrm{in}$ can be calculated by equation (S2); $N_{\mathrm{hydrat}e}$ can be calculated by equation (S4), and before that, the ice conversion ratio $x_t$ should be calculated by equation (S6). Then, $N_{\mathrm{gas,t}}$ can be calculated based on equation (S1). The compressibility factors used in the equation of state to calculate of $N_\mathrm{in}$ and $N_{\mathrm{gas,t}}$ belonged to corresponding gas mixtures.

The recovery ratio of component $i$ is calculated by

$$R_i = \frac{N_{\text{gas}} y_i}{N_{\text{in}} z_i} \tag{S13}$$

## Ⅱ. ***Characterization***

(1) *Raman spectra characterization.*

The Raman spectroscopy tests were carried out using a HORIBA XploRA multichannel fibre optic-based system. A 532 *nm* wavelength laser with 1800 grooves $mm^{-1}$ grating was used. The spectral resolution was 1 $cm^{-1}$. The optical reactor as depicted in Supplementary Fig. 14 was used for in situ hydrate formation and dissociation in the tests. Before the experiments, the reactor was washed with deionized water and dried. Subsequently, 1.5 g of SDS solution was poured into the reactor. Then, the reactor was cooled to 274.15 K. The reactor was vacuumed and 9.6 MPa methane gas was injected into the reactor. 6 *h* was given for hydrate formation to let the water convert into gas hydrate as much as possible. Subsequently, the temperature of the reactor was adjusted to 272.15 K to cool the hydrate. When the temperature of the hydrate reached the desired value, the reactor was depressurized to atmospheric pressure quickly. The hydrate dissociated to the active ice below the ice point and under atmospheric pressure. Subsequently, 9.6 MPa methane was reinjected into the reactor to form hydrate in the active ice. The changes in Raman spectroscopy were determined before and after hydrate formation in the active ice.

(2) *DSC.*

The decomposition enthalpy and the melting temperature of the active ice were measured with a high pressure micro-differential scanning calorimeter (HP μ-DSC7 Evo, Setaram Inc, France). The HP μ-DSC7 has one sample and one reference cell, both of which have an effective volume of 0.5 ml. The HP μ-DSC7 has a resolution of 0.02 μW and a temperature deviation of ±0.2 K. Before each experiment, the sample cell was washed with deionized water and dried. 0.05 g SDS solution was added to the sample cell. The sample cell was mounted into the calorimeter and sealed tightly. To accurately measure the mass of water in the SDS solution, the sample cell was first cooled to 253.15 K to completely freeze the SDS solution, and then heated to 283.15 K at a rate of 0.5 K $min^{-1}$ to melt the ice. The accurate mass of water in the loaded SDS solution can be calculated from the total heat absorption during the ice melting and the ice melting enthalpy of 6.01 kJ $mol^{-1}$. After that, the sample cell was

pressurized with methane to 9.6 MPa. To make the SDS solution convert into gas hydrate as much as possible, the temperature was programmed to fluctuate between 275.15 K and 258.15 K with a rate of 1 K $min^{-1}$ (Supplementary Fig. 16). Subsequently, the melting temperature and the melting enthalpy of the active ice were measured by heating it to 298.15 K at a rate of 0.25 K $min^{-1}$.

To investigate if some SDS solution is unfrozen under the ice point, approximately 0.0370 g SDS solution was injected into the sample cell, and the mass of the SDS solution was precisely measured by a balance with accuracy of 0.0001 g. The temperature of the sample cell was decreased to 253.15 K and kept for 15 minutes, and then increased to 278.15 K with heating rate of 1 K $min^{-1}$. This procedure was repeated two times to freeze the solution as much as possible. Subsequently, the solution was cooled to 253.15 K and kept for 1 h, and then heated to 278.15 K with heating rate of 0.25 K. The amount of frozen solution was calculated by dividing the measured heat of fusion by the heat of fusion of unit amount of ice (6.01 kJ $mol^{-1}$). Then the unfrozen ratio of the solution could be calculated by the amount of frozen solution and the total amount of the solution that measured by the balance.

(3) *cryo-SEM characterization*.

A Hitachi SU3500 scanning electron microscope was used for the characterization. A Quorum PP3000T cryo-SEM preparation system was used for keeping the active ice stable during the characterization. The active ice was not directly prepared in the preparation system. When the active ice preparation in the sapphire reactor via hydrate formation−dissociation method was accomplished, the sapphire reactor was cooled to 268.15 K for 3 hours. Subsequently, the active ice was taken out from the reactor, and was put into a sample container, which was precooled with liquid nitrogen. Afterwards, one piece of the active ice was chosen and broken off under liquid nitrogen atmosphere. Then, one of the fragments was transferred to the SEM cryo-stage for imaging. Because the sample was almost ice, it did not experience sublimation and coating before the transfer to avoid affecting the appearance of the active ice. The temperature of the cryo-stage was kept at 133.15 K. The SEM image of the fragment was obtained at atmospheric pressure and 5 kV of accelerating voltage.

(4) *PXRD characterization*.

PXRD characterization on the active ice and the other ice powders were conducted with a Malvern Panalytical X-ray diffractometer (Empyrean range). The active ice was grinded under dry ice atmosphere. Then the grinded powder was placed onto a steel substrate, which was precooled by dry ice. The substrate with sample was mounted in the PXRD, then the spectrum was taken immediately. The scanning was conducted under room temperature and before the melting of the active ice. The PXRD characterization on the other ice powders were conducted with the same procedure.

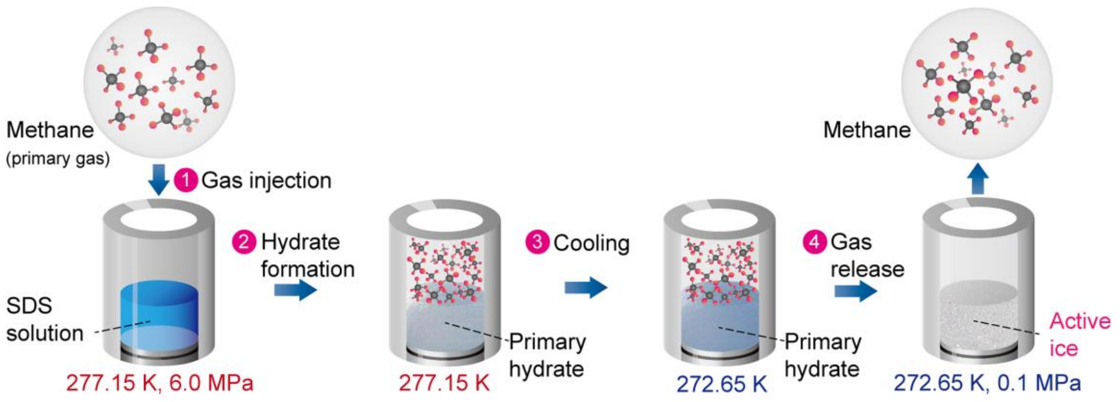


**Supplementary Fig. 1. Schematic of the active ice preparation.**

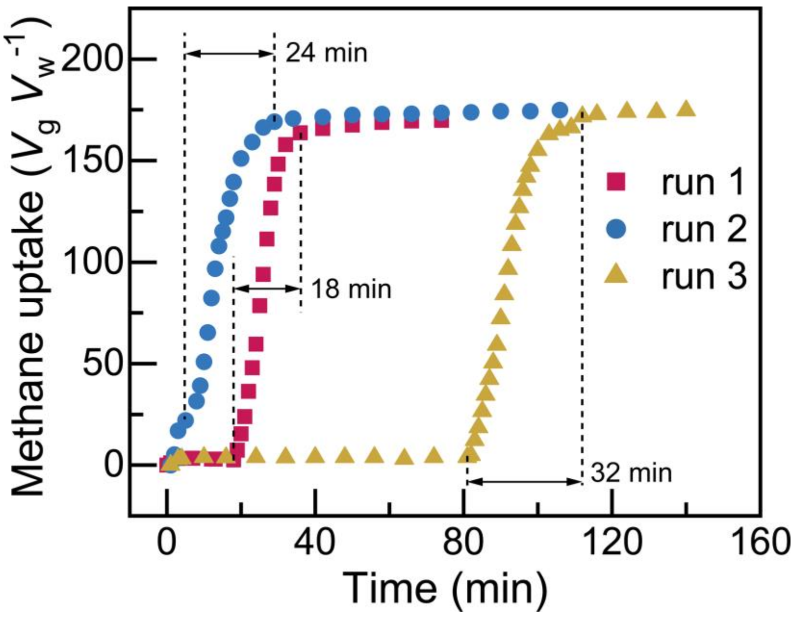


**Supplementary Fig. 2. Methane uptake in 600-ppm SDS solutions at 272.65 K and initial pressure of 6.0 MPa.** The induction time for runs 1-3 were 18, 5, and 82 mins, respectively. Stirring was adopted to accelerate hydrate nucleation, and it was switched off since hydrate appeared in the reactor.

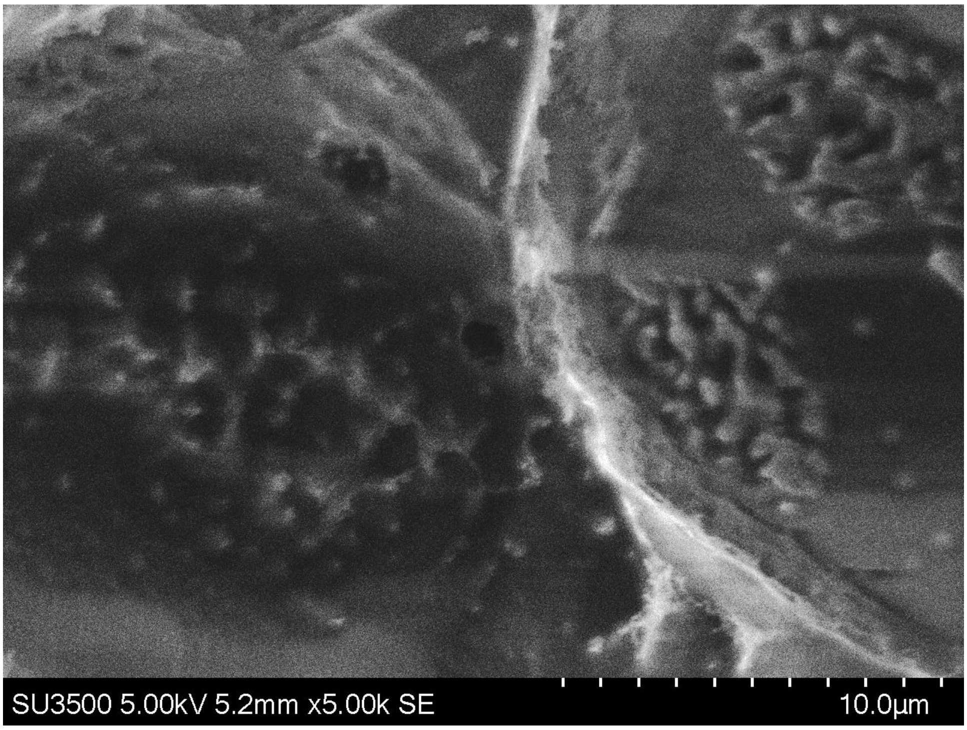


**Supplementary Fig. 3. Scanning electron micrograph of the active ice showing the porous structure and the bud like protuberances in the pores.**

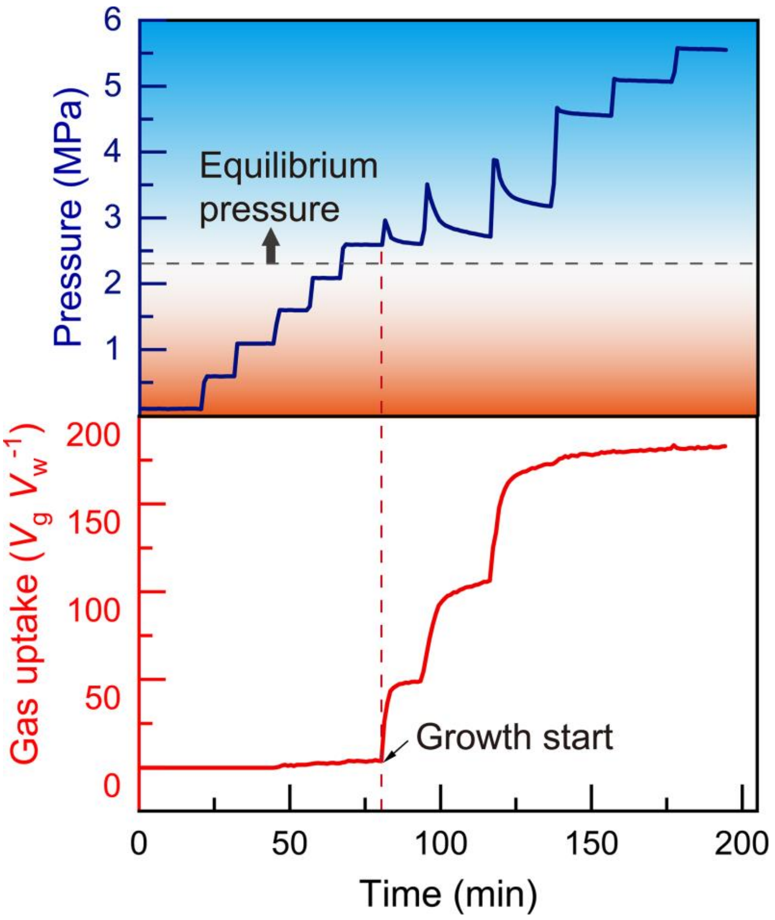


**Supplementary Fig. 4. The pressure change and gas uptake in 10.0 g active ice over step-by-step pressurization.** The active ice was prepared by dissociating $CH_4$−SDS hydrate below the ice point. The temperature during the pressurization was fixed at 272.65 K.

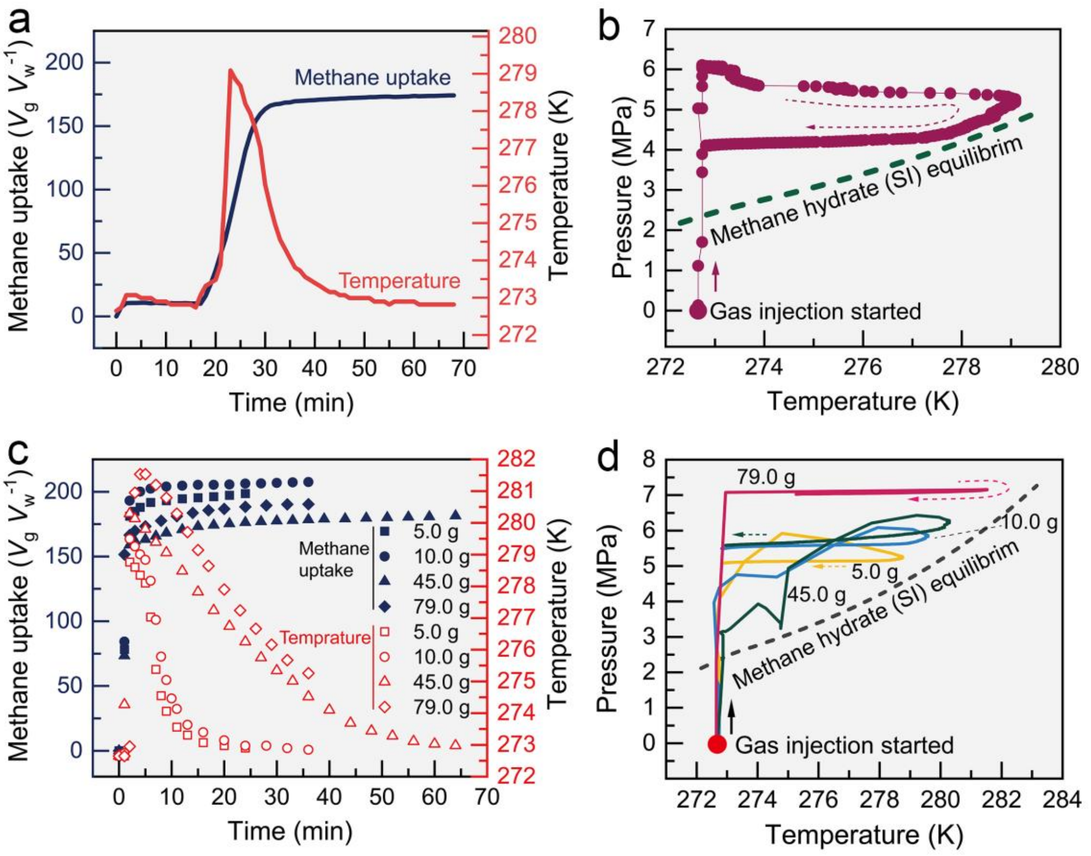


**Supplementary Fig. 5. Comparison of methane hydrate formation in SDS solution and active ice of different masses.** The experiments of hydrate formation in 10.0 g 600-ppm SDS solution and in 5.0 g, 10.0 g, 45.0 g active ice were conducted at 272.65 K and initial pressure of 6.0 MPa. For 79.0 g active ice, the pressure was kept at 7.0 MPa. a and b Gas uptake and temperature change (a) and temperature−pressure change (b) during hydrate formation in 10.0 g SDS solution. c and d Gas uptake and temperature change (c) and temperature−pressure change (d) during methane hydrate formation in active ice packing beds with different ice masses. The active ice was grinded into 180 ~ 250 μm and naturally packed. The sizes were φ2.54×2.00, φ2.54×4.10, φ5.10×4.50, and φ5.10×7.95 cm for the packing beds with ice mass of 5.0, 10.0, 45.0, and 79.0 g, respectively. The experiments with 5.0 g and 10.0 g active ice were conducted in the sapphire reactor depicted in Supplementary Fig. 13, while the experiments with 45.0 g and 79.0 g active were conducted in a larger stainless-steel reactor (234 ml). In the experiments with 5.0, 10.0 and 45.0 g active ice, each point of the T−P curve was obtained every 5 seconds; when 79.0 g active ice was adopted, it was obtained every 1 minute.

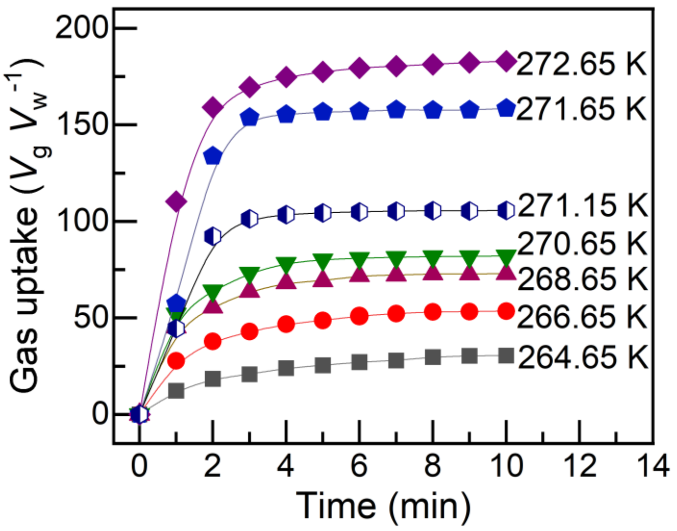


**Supplementary Fig. 6. Gas uptake of methane hydrate formed in the active ice at different temperatures and initial pressure of 6.0 MPa.**

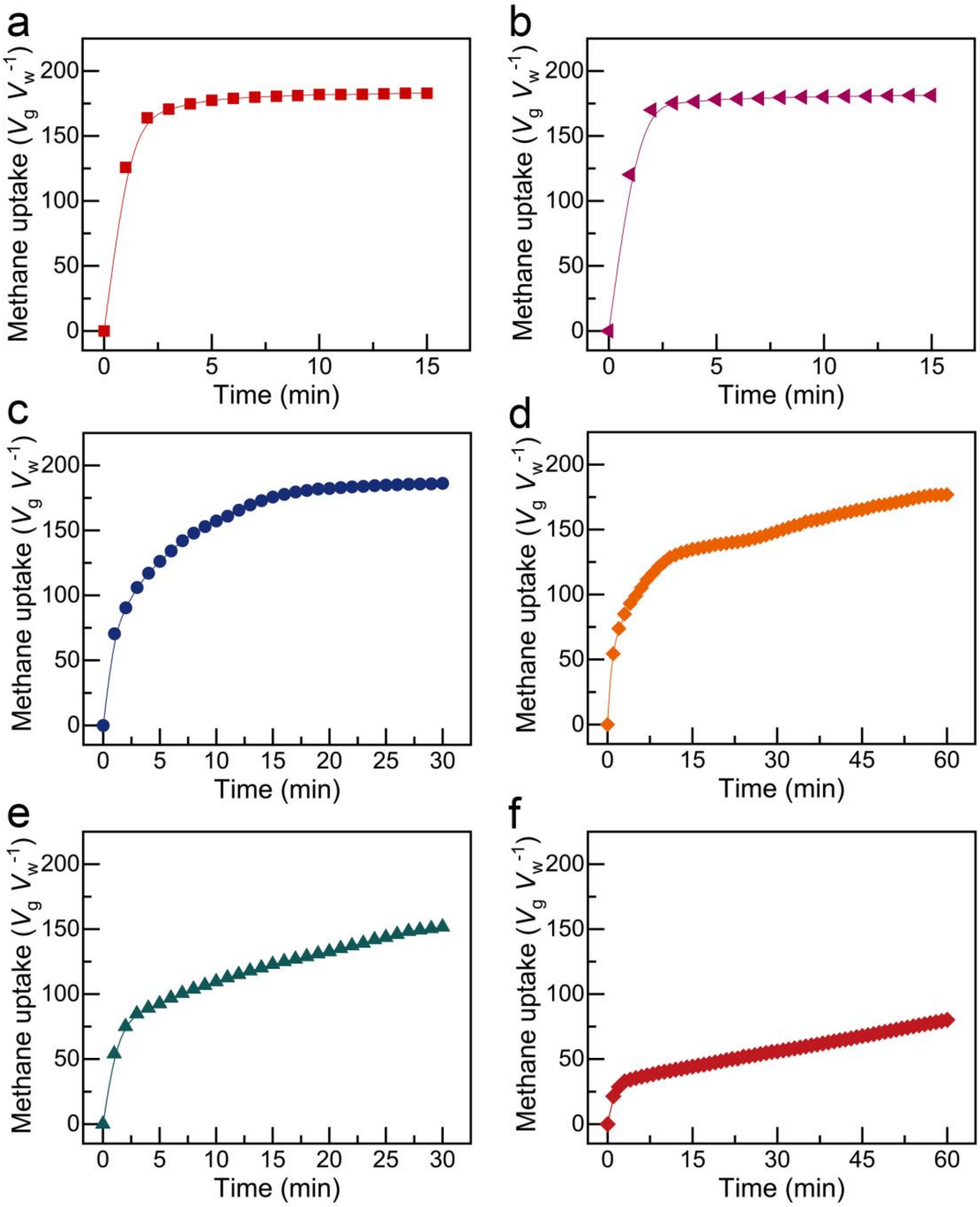


**Supplementary Fig. 7. Gas uptake of methane hydrate formed in active ices prepared in the presence of different kinetic promoters.** a 600-ppm sodium dodecanoate. b 600-ppm sodium dodecyl benzene sulfonate. c 600-ppm dodecylbenzenesulfonic acid. d 600-ppm sodium laurylsulfonate. e 600-ppm sodium oleate. f 600-ppm N-Carbobenzoxy-DL-leucine.

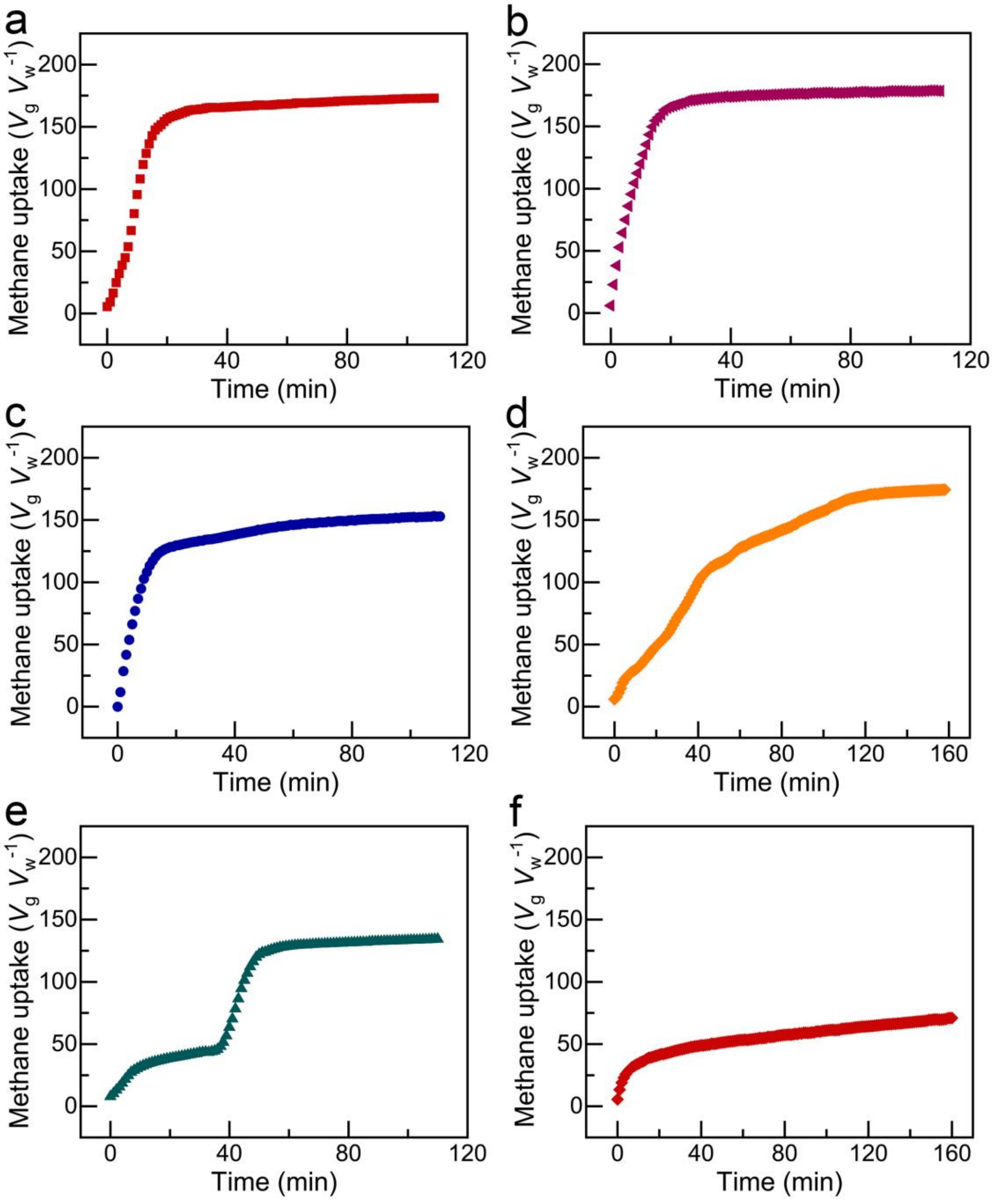


**Supplementary Fig. 8. Gas uptake of methane hydrate formed in different kinetic promoter solutions at 272.65 K and initial pressure of 6.0 MPa.** a 600-ppm sodium dodecanoate. b 600-ppm sodium dodecyl benzene sulfonate. c 600-ppm dodecylbenzenesulfonic acid. d 600-ppm sodium laurylsulfonate. e 600-ppm sodium oleate. f 600-ppm N-Carbobenzoxy-DL-leucine. The induction times are not shown in the figures, therefore the methane uptake curves do not start at 0. The induction times for figures a-f are 39, 12, 1, 7, 282, 4 min, respectively.

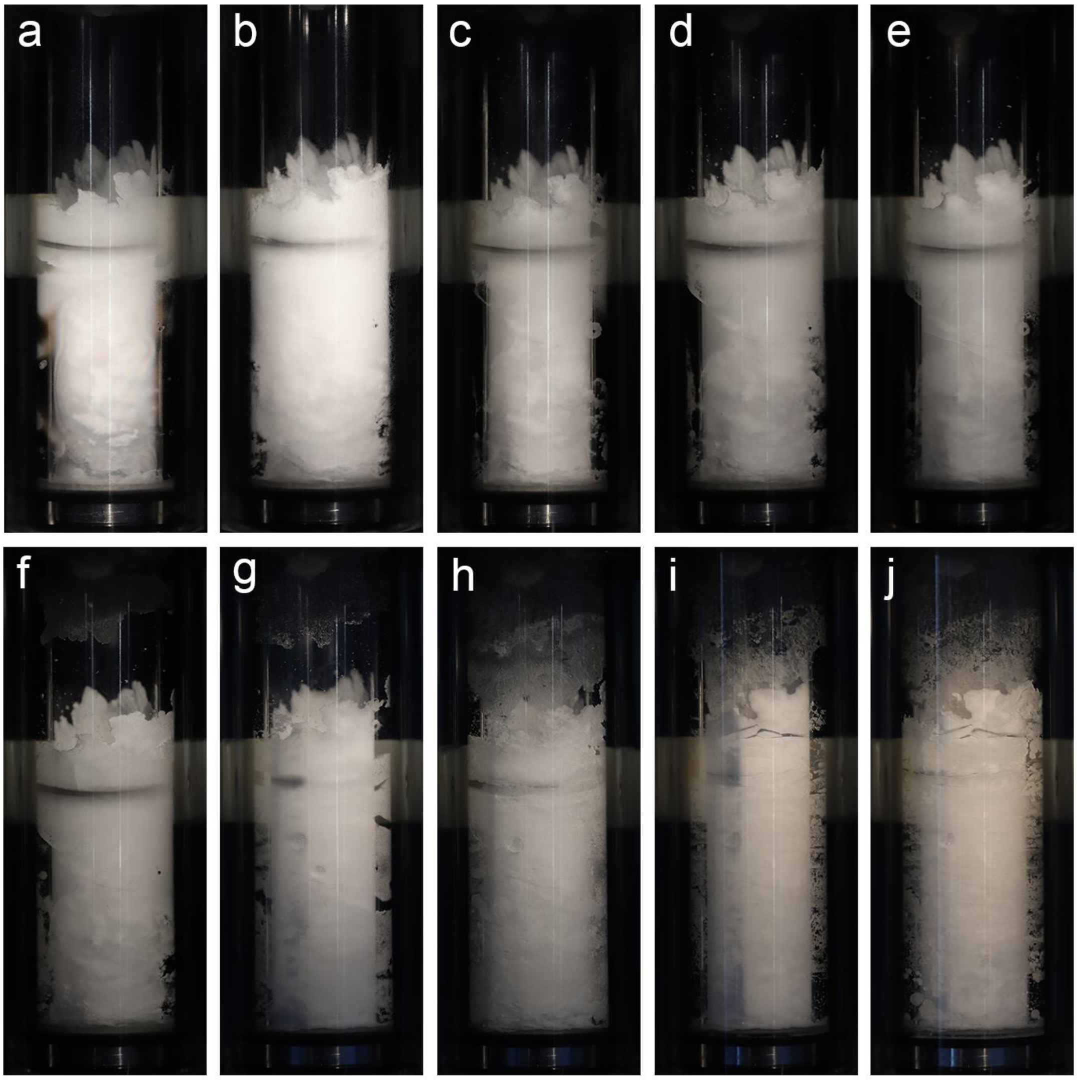


**Supplementary Fig. 9. Sequential images of the active ice and methane hydrate in cycle experiments of methane hydrate formation and dissociation.** (a and b) Images of the active ice and methane hydrate in the $1^{st}$ cycle, (c and d) $2^{nd}$ cycle, (e and f) $3^{rd}$ cycle, (g and h) $4^{th}$ cycle, and (i and j) $5^{th}$ cycle.

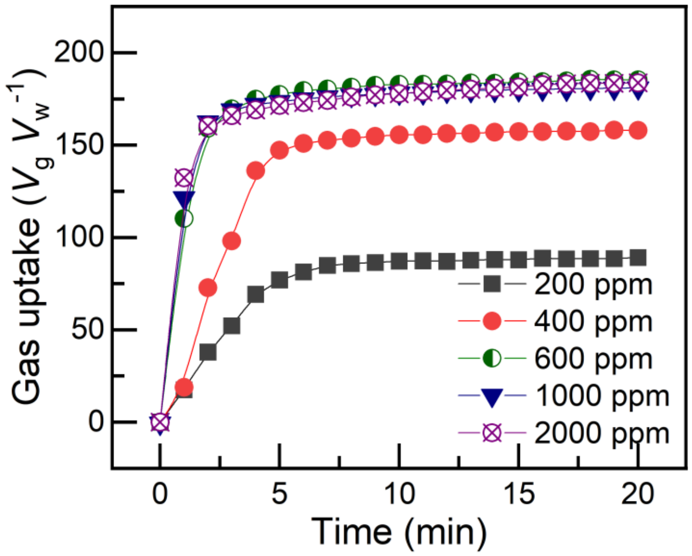


**Supplementary Fig. 10. Methane uptake in the active ices with different SDS concentrations.** The hydrate formation experiments were conducted at 272.65 K and initial pressure of 6.0 MPa.

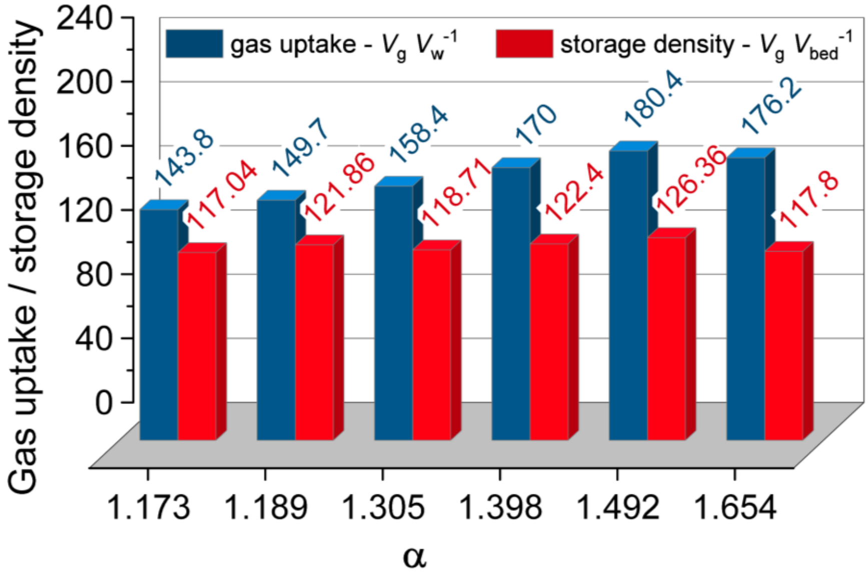


**Supplementary Fig. 11. Methane uptake and storage density in the compressed active ice columns with different loosening coefficients.** The mass of the active ice was 10.0 g, and the data were acquired at 9 min in each hydrate formation experiment. The temperature and initial pressure for hydrate formation were 272.65 K and 6.0 MPa, respectively. Free gas in the packing bed is included in the storage density.

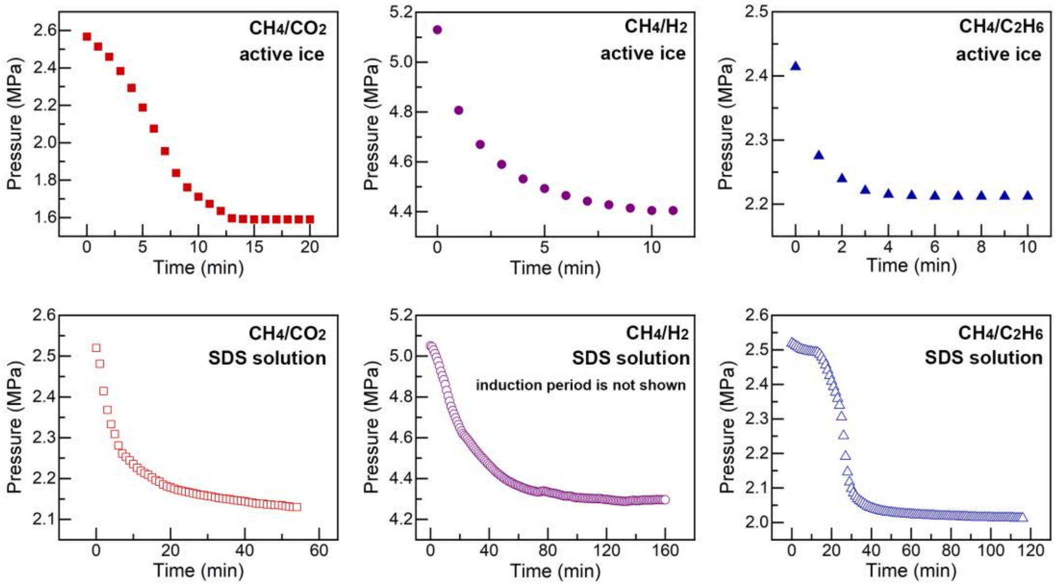


**Supplementary Fig. 12. Pressure change in the gas separation experiments with 10.0 g active ice or SDS solution.** The temperature was fixed at 272.65 K; the mass concentration of SDS in both the active ice and solution were 600 ppm.

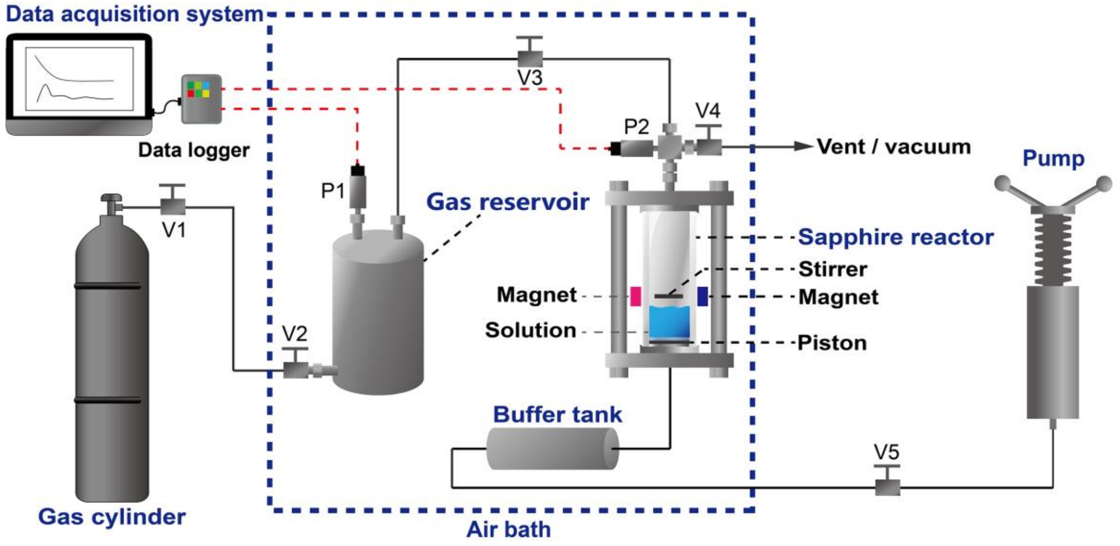


**Supplementary Fig. 13. Schematic of the apparatus for preparing and utilizing the active ice.** V1~V5, needle valves; P1 and P2, pressure sensors.

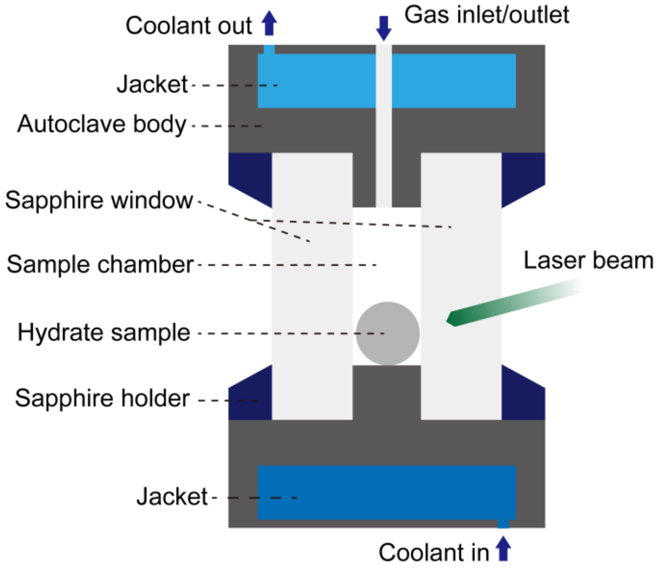


**Supplementary Fig. 14. Schematic of the in situ optical reactor for Raman spectra characterization.**

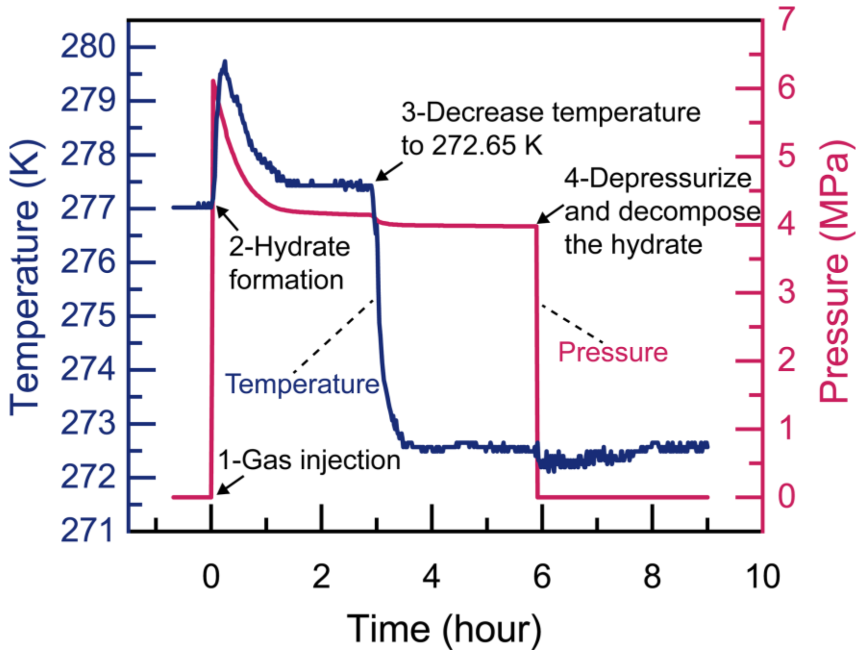


**Supplementary Fig. 15. Temperature and pressure change over time in preparing the active ice via dissociating methane hydrate below the ice point.** The temperature sensor was covered by methane hydrate after hydrate formation finished, otherwise, the temperature when depressurizing the reactor would reduce by lower than 10 K because of Joule-Thomson effect.

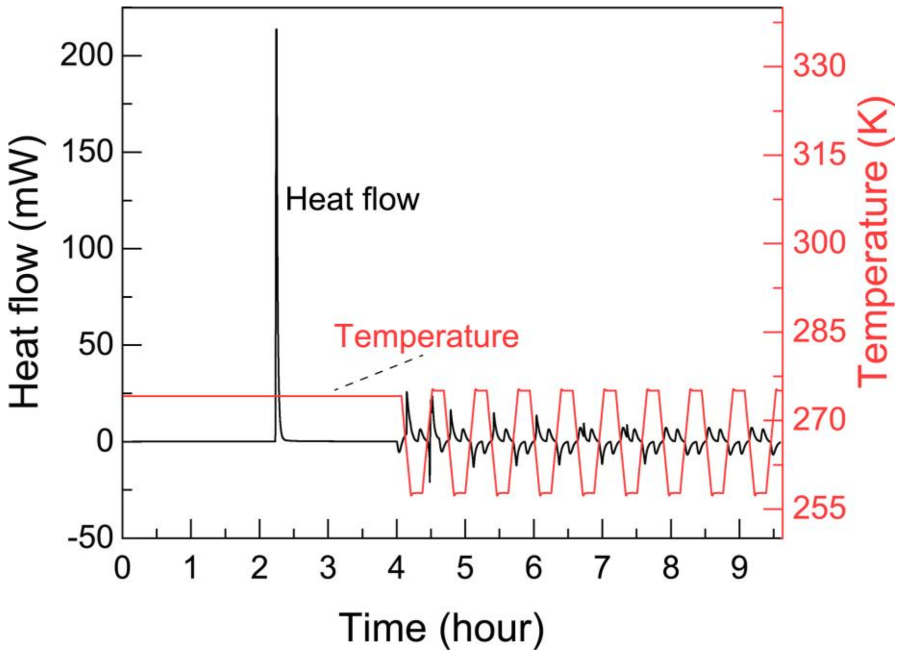


**Supplementary Fig. 16. Temperature and heat flow profiles during methane hydrate formation in the micro-differential scanning calorimeter.** The upward heat peaks indicate exothermic hydrate formation and downward heat peaks indicate endothermic melting of ice. The water completely converted into methane hydrate after 8 h, and the subsequent heat flow fluctuation was due to temperature change.

**Supplementary Table 1.**
**Thermal property measurements for the active ice and aqueous ice.**

| Substance | Melting temperature (K) | Enthalpy (kJ $mol^{-1}$) |
|---|---|---|
| active ice | 273.29 | 5.990 |
| aqueous ice | 273.23 | 6.023 |

**Supplementary Table 2.**

**Melting temperature and latent heat of frozen SDS solution with different concentrations, showing unfrozen water exists in the ice containing SDS.** The solutions were cooled to 253.15 K and kept for 1 h, and then heated to 278.15 K with heating rate of 0.25 K to measure the heat absorbed during ice melting.

| Mass concentration of SDS | 0.40% | 5.09% | 9.83% |
| --- | --- | --- | --- |
| Mass of solution (g) | 0.0370 | 0.0362 | 0.0370 |
| Melting temperature (K) | 273.17 | 273.24 | 273.23 |
| Latent heat (mJ) | $1.24\times10^4$ | $1.14\times10^4$ | $1.09\times10^4$ |
| Ratio of unfrozen water | 0.56% | 0.84% | 2.37% |

**Supplementary Table 3.**

**Comparison of gas separation via the active ice and 600-ppm SDS solution*.**

| Gas mixture | Separation medium | Initial pressure (MPa) | Final pressure (MPa) | $R_{g\text{-}m}$[a] | Induction time (min) | Formation time (min) | $y_1$[b] | $y_2$[b] | $x_1$[b] | $x_2$[b] | $R_2$ (%) | $\beta$ |
|---|---|---|---|---|---|---|---|---|---|---|---|---|
| $CH_4$(*1*)/$CO_2$(*2*) (0.482:0.518) | active ice | 2.57 | 1.59 | 127.46 | NO[c] | 20 | 0.592 | 0.408 | 0.265 | 0.735 | 48.03 | 4.02 |
| (0.458:0.542) | SDS solution | 2.52 | 2.15 | 152.45 | NO[c] | 54 | 0.500 | 0.500 | 0.295 | 0.705 | 26.72 | 2.39 |
| $H_2$(*1*)/$CH_4$(*2*) (0.255:0.745) | active ice | 5.13 | 4.40 | 304.57 | NO[c] | 11 | 0.355 | 0.645 | 0.002 | 0.998 | 38.22 | 355.66 |
| | SDS solution | 5.12 | 4.30 | 342.90 | 936 | 160 | 0.327 | 0.673 | 0.008 | 0.992 | 29.87 | 59.11 |
| $CH_4$(*1*)/$C_2H_6$(*2*) (0.940:0.060) | active ice | 2.41 | 2.21 | 110.73 | NO[c] | 10 | 0.985 | 0.015 | 0.701 | 0.299 | 78.55 | 27.47 |
| | SDS solution | 2.52 | 2.01 | 134.02 | 16 | 100 | 0.985 | 0.015 | 0.774 | 0.226 | 80.68 | 18.89 |

[*] the amounts of the active ice and the SDS solution were fixed to 10.0 g, and the temperature was 272.65 K;
[a] volumetric ratio of gas (standard temperature and pressure) to materials (SDS solution or active ice); because the volume of the solution is smaller than the volume of equivalent active ice, this ratio was smaller when the active ice was used;
[b] equilibrium composition of gas phase ($y$) and hydrate phase ($x$);
[c] not observed.
